# Supplementary material for: HybriSeq: probe-based device-free single-cell RNA profiling
Source: Commun Biol. 2025 Aug 19;8:1250. doi: 10.1038/s42003-025-08702-8 (PMC12365068; doi:10.1038/s42003-025-08702-8)
Supplement: Supplementary file 2 — Description of Additional Supplementary Files [file 42003_2025_8702_MOESM2_ESM.docx]

**Description of Additional Supplementary Files**

File name: Supplementary Data 1

Description: cell cycle mouse human probe

File name: Supplementary Data 2

Description: Tile probes

File name: Supplementary Data 3

Description: ENCODE data ref

File name: Supplementary Data 4

Description: PBMC probes

File name: Supplementary Data 5

Description: cost

File name: Supplementary Data 6

Description: linker quench oligos

File name: Supplementary Data 7-9

Description: round 1-3 BC

File name: Supplementary Data 10

Description: qPCR probes, primers, curves
