# Supplementary material for: HybriSeq: probe-based device-free single-cell RNA profiling
Source: Commun Biol. 2025 Aug 19;8:1250. doi: 10.1038/s42003-025-08702-8 (PMC12365068; doi:10.1038/s42003-025-08702-8)
Supplement: Supplementary file 4 — Reporting summary [file 42003_2025_8702_MOESM4_ESM.pdf]

nature portfolio

Corresponding author(s): Bo Huang

Last updated by author(s): Aug 4, 2025

Reporting Summary

Nature Portfolio wishes to improve the reproducibility of the work that we publish. This form provides structure for consistency and transparency in reporting. For further information on Nature Portfolio policies, see our Editorial Policies and the Editorial Policy Checklist.

Statistics

For all statistical analyses, confirm that the following items are present in the figure legend, table legend, main text, or Methods section.

n/a	Confirmed	
☐	☒	The exact sample size (*n*) for each experimental group/condition, given as a discrete number and unit of measurement
☒	☐	A statement on whether measurements were taken from distinct samples or whether the same sample was measured repeatedly
☐	☒	The statistical test(s) used AND whether they are one- or two-sided<br>*Only common tests should be described solely by name; describe more complex techniques in the Methods section.*
☒	☐	A description of all covariates tested
☐	☒	A description of any assumptions or corrections, such as tests of normality and adjustment for multiple comparisons
☒	☐	A full description of the statistical parameters including central tendency (e.g. means) or other basic estimates (e.g. regression coefficient) AND variation (e.g. standard deviation) or associated estimates of uncertainty (e.g. confidence intervals)
☐	☒	For null hypothesis testing, the test statistic (e.g. *F*, *t*, *r*) with confidence intervals, effect sizes, degrees of freedom and *P* value noted<br>*Give P values as exact values whenever suitable.*
☒	☐	For Bayesian analysis, information on the choice of priors and Markov chain Monte Carlo settings
☒	☐	For hierarchical and complex designs, identification of the appropriate level for tests and full reporting of outcomes
☒	☐	Estimates of effect sizes (e.g. Cohen's *d*, Pearson's *r*), indicating how they were calculated

Our web collection on statistics for biologists contains articles on many of the points above.

Software and code

Policy information about availability of computer code

Data collection	Miseq runs used Illumina RTA 1.18.54.4
Data analysis	custom Python (3.7.4) code was used for analysis and plot generation, scanpy 1.9.3 was used for single cell analysis, samtools 1.10 for long read RNA seq analysis, bowtie2 2.3.5.1 for probe alignment, QuantStudio™ Design and Analysis Software v1.4.1 was used for qPCR, Salmon 0.12.0 was used for RNAseq analysis

For manuscripts utilizing custom algorithms or software that are central to the research but not yet described in published literature, software must be made available to editors and reviewers. We strongly encourage code deposition in a community repository (e.g. GitHub). See the Nature Portfolio guidelines for submitting code & software for further information.

Data

Policy information about availability of data

All manuscripts must include a data availability statement. This statement should provide the following information, where applicable:

- Accession codes, unique identifiers, or web links for publicly available datasets
- A description of any restrictions on data availability
- For clinical datasets or third party data, please ensure that the statement adheres to our policy

A public git repository is available at https://github.com/BoHuangLab/HybriSeq containing source code and a minimum analytical dataset sufficient to reproduce

figures in the main text. This repository is also available through Figshare (https://doi.org/10.6084/m9.figshare.23882193.v2). The fastq files and processed data are available through GEO (GSE292322)

Research involving human participants, their data, or biological material

Policy information about studies with human participants or human data. See also policy information about sex, gender (identity/presentation), and sexual orientation and race, ethnicity and racism.

Reporting on sex and gender	NA
Reporting on race, ethnicity, or other socially relevant groupings	NA
Population characteristics	NA
Recruitment	NA
Ethics oversight	NA

Note that full information on the approval of the study protocol must also be provided in the manuscript.

Field-specific reporting

Please select the one below that is the best fit for your research. If you are not sure, read the appropriate sections before making your selection.

☒ Life sciences ☐ Behavioural & social sciences ☐ Ecological, evolutionary & environmental sciences

For a reference copy of the document with all sections, see nature.com/documents/nr-reporting-summary-flat.pdf

Life sciences study design

All studies must disclose on these points even when the disclosure is negative.

Sample size	no sample size calculation was performed, many cells but only single samples were used to generate the data. Cell number used were determined by limitations of available cells or method limitations.
Data exclusions	no data was excluded from analysis
Replication	Findings were not replicated
Randomization	not relevant
Blinding	not relevant

Reporting for specific materials, systems and methods

We require information from authors about some types of materials, experimental systems and methods used in many studies. Here, indicate whether each material, system or method listed is relevant to your study. If you are not sure if a list item applies to your research, read the appropriate section before selecting a response.

Materials & experimental systems

n/a	Involved in the study
☒	☐ Antibodies
☐	☒ Eukaryotic cell lines
☒	☐ Palaeontology and archaeology
☒	☐ Animals and other organisms
☒	☐ Clinical data
☒	☐ Dual use research of concern
☒	☐ Plants

Methods

n/a	Involved in the study
☒	☐ ChIP-seq
☒	☐ Flow cytometry
☒	☐ MRI-based neuroimaging

Eukaryotic cell lines

Policy information about cell lines and Sex and Gender in Research

Cell line source(s)	HEK293 cells were originally obtained from the UCSF Cell Culture Facility. HeLa (CCL-2) cells were originally obtained from ATCC. Do-11-10 cells from Sigma-Aldrich 85082301. PBMC were purchased from Cytologics.
Authentication	no authentication
Mycoplasma contamination	cells were not tested for mycoplasma contamination
Commonly misidentified lines (See ICLAC register)	HEK293 cells used as cells in optimization, identity not crucial to findings.

Plants

Seed stocks	NA
Novel plant genotypes	NA
Authentication	NA
